# A skin-inspired durable de-icing surface with boosting interfacial cracks

**DOI:** 10.1093/nsr/nwaf005

**Published:** 2025-01-13

**Authors:** Qiucheng Yang, Jinlong Yang, Yuhao Hu, Xiaopeng Niu, Zhenda Liu, Jian Zou, Junchang Guo, Hao Xiong, Xingshi Gu, Li Yang, Fanfei Yu, Shunpeng Zhu, Ming Ye, Xian Yi, Xu Deng

**Affiliations:** Institute of Fundamental and Frontier Sciences, University of Electronic Science and Technology of China, Chengdu 611731, China; Institute of Fundamental and Frontier Sciences, University of Electronic Science and Technology of China, Chengdu 611731, China; Institute of Fundamental and Frontier Sciences, University of Electronic Science and Technology of China, Chengdu 611731, China; School of Mechanical and Electrical Engineering, University of Electronic Science and Technology of China, Chengdu 611731, China; Institute of Fundamental and Frontier Sciences, University of Electronic Science and Technology of China, Chengdu 611731, China; School of Materials and Energy, University of Electronic Science and Technology of China, Chengdu 611731, China; Institute of Fundamental and Frontier Sciences, University of Electronic Science and Technology of China, Chengdu 611731, China; State Key Laboratory of Aerodynamics, Mianyang 621000, China; State Key Laboratory of Aerodynamics, Mianyang 621000, China; Institute for Advanced Study, Chengdu University, Chengdu 610106, China; Department of Mechanical Engineering, The Hong Kong Polytechnic University, Hong Kong 999077, China; School of Mechanical and Electrical Engineering, University of Electronic Science and Technology of China, Chengdu 611731, China; Bruker nano surface and metrology, Santa Barbara, CA 93117, USA; State Key Laboratory of Aerodynamics, Mianyang 621000, China; Institute of Fundamental and Frontier Sciences, University of Electronic Science and Technology of China, Chengdu 611731, China; Shenzhen Institute for Advanced Study, University of Electronic Science and Technology of China, Shenzhen 518110, China

**Keywords:** interfacial phenomena, de-icing, ice adhesion, wettability

## Abstract

Fracture-based interfacial breakage has shown promise in efficiently removing ice accretion. Here, intrigued by the response of human skin to stress-induced deformation, we present a strategy to design tough-skin de-icing surfaces (TSDSs) that actively manipulate crack-induced ice-substrate interfacial breakage during ice removal. This design leverages the surface instability of thin films to generate extensive wrinkling at the ice-substrate interface, which serves as crack initiation sites. We demonstrate efficient ice shedding by creating wrinkles at two length scales: macro-wrinkles for actively initiating the cracks at the rim of the ice and micro-wrinkles for further promoting the stress concentration at the ice-substrate interface. The TSDS (*τ* < 10 kPa) displays excellent durability and weather resistance, achieving a large-area ice-self-shedding effect solely through gravity. The universality of the proposed mechanism is verified on multiple materials and potential applications. This design concept offers valuable insights into the creation of durable de-icing materials with enhanced ice-shedding properties.

## Teaser

This work introduces a durable de-icing surface inspired by human skin, using wrinkles at different sizes to help the ice to break away more easily from the surface.

## INTRODUCTION

Ice accretion tightly adheres to surfaces due to strong interaction forces such as van der Waals force, electrostatic force and hydrogen bonds between them [1,2]. This significantly complicates de-icing efforts across various fields, including transportation systems [3,4], power systems [5] and infrastructure [6,7]. From an interfacial perspective, the primary challenge lies in effectively disrupting the ice-solid interface and therefore removing ice accumulations that cling to various surfaces. This issue underscores the necessity for efficient methods to fracture the ice-solid interface and reduce ice adhesion.

**Figure 1.**
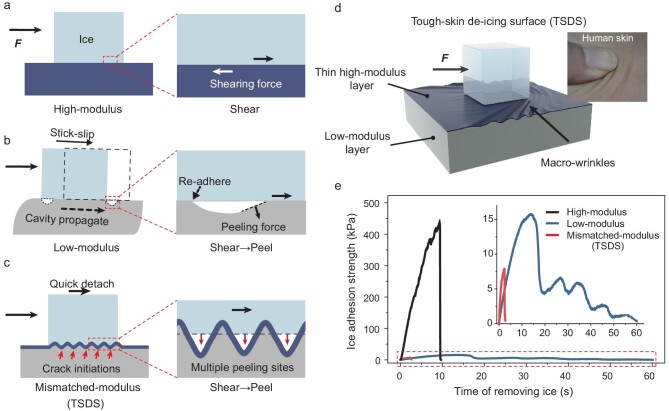
Design strategy of the TSDS. (a) and (b) Schematics of de-icing processes from the high-/low-modulus surface by shear stress. (c) Deformation of the mismatched-modulus surface as a shear force applied on the ice. In the enlarged image on the right, deformation causes the transformation from shear stress into peel stress at localized regions, resulting in multiple peeling sites generated at the ice-solid interface. (d) 3D schematic diagram of the de-icing process on the TSDS. (e) Ice-adhesion strength measured for three types of surfaces.

For conventional structural substrates, their inherent rigidity prevents surface deformation under shearing forces (Fig. 1a). This leads to a state of pure shear stress at the surface. The strong interaction between ice and substrate requires significant energy to remove the ice accretion. Typical hard structural surfaces, such as aluminum and steel, exhibit ice-adhesion strengths of >1000 kPa [1]. Recent strategies for reducing ice adhesion, including those inspired by nature [8–12], have focused on superhydrophobic surfaces to minimize the ice-solid contact [13–17] and liquid-infused surfaces to introduce a thin fluid layer between the ice and the substrate [18–22]. However, these methods may present new challenges: superhydrophobic surfaces are structurally fragile, while liquid-infused surfaces risk losing their liquid medium.

An alternative effective way to reduce ice adhesion is to introduce a soft layer that promotes cavitation at the interface [23–26]. From the viewpoint of fracture mechanics, the ice-adhesion strength can be estimated by


(1) \begin{eqnarray*}
{\tau _{{\mathrm{ice}}}} = \sqrt {\frac{{E^*G}}{{\pi l \Lambda }}},
\end{eqnarray*}

where E* is the apparent elastic modulus, *G* is the material surface energy, *l* is the crack length on the ice–solid interface and *ᴧ* is a non-dimensional constant that is affected by the geometric configuration of the crack [23]. In terms of the stress state, shear stress transforms into a peeling force due to deformation of the soft substrate at the front end of the cavity (Fig. 1b). The peeling force enables the separation of two adhered objects with less effort compared with shearing force, resulting in a significant reduction in the ice adhesion (Video S1 and Table S1). Additionally, the deformation of the elastic material promotes the generation of more crack sources at the ice-solid interface, which increases *l*. The soft property of the substrate facilitates the ice shedding, yet removing ice from a surface with a low modulus may lead to stick-slip motion (Fig. 1b and Video S2), as evidenced by the fluctuating shearing curve shown in Fig. 1e. The Schallamach wave, associated with stick-slip movement, nucleates due to the buckling instability of the elastic surface [27]. During the de-icing process, trapped air cavities propagate as pulses along the ice-solid interface. The detached interface reattaches, causing the ice to slip continuously along the surface [28]. For surfaces with a modulus that is too low, the stick-slip motion increases the energy consumption for de-icing and the low mechanical properties limit their application, especially in harsh service environments.

Increasing the crack length or number of crack initiation sites could effectively reduce ice-adhesion strength, but achieving this is challenging. When water freezes and transitions from liquid to solid, the ice shape adapts to the morphology of the solid surface, causing cracks to be eliminated during the phase-change process. Consequently, the challenge persists in designing and fabricating durable de-icing materials with crack sources that perform reliably under practical conditions. Recent attempts to use prefab cavitation by integrating rigid components into soft layers [24] or the soft phase is wrapped in the hard phase [23,25,26] have shown promise in reducing ice-removal energy. However, these methods are constrained by limited crack initiation sites and reduced durability in practical applications. The design and fabrication of durable de-icing materials that function effectively under harsh conditions remain significant challenges in this field.

Here, we present a design strategy and a versatile approach to actively transform the stress state at the ice-solid interface by creating a tough-skin de-icing surface (TSDS). This design features a modulus mismatch between a thin, hard top film and a soft substrate (Fig. 1c and d). Inspired by human skin, in which the soft dermis and subcutaneous tissue are encased by the protective epidermis, our TSDS mimics this natural structure. When subjected to external forces, skin deforms and folds (Fig. 1d). Similarly, in our design, wrinkles form on the surface due to the instability of the tough skin under the shear stress exerted by the ice. These wrinkles initiate cracks at the interface and the high modulus of the top film promotes brittle fracture, effectively preventing the stick-slip effect that is typically caused by the soft substrate. The outcome significantly reduces the energy required for ice removal by effectively managing disruptions at the ice-solid interface (Fig. 1e). We show the mechanism of the wrinkles-induced interfacial crack boosting in two length scales. The tough-skin coating exhibits ice adhesion of <10 kPa. Leveraging the hard skin system, this de-icing material exhibits excellent durability across a series of tests. Finally, we demonstrate the applications of this strategy for mitigating the impacts of ice accretion in various systems.

## RESULTS AND DISCUSSION

### Design strategy of TSDS

**Figure 2.**
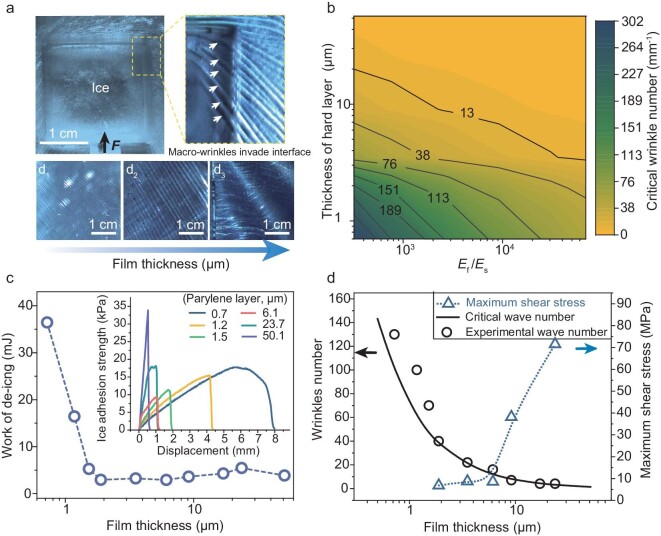
Mechanism of wrinkle generation on the TSDS and impact on ice-adhesion strength. (a) Photograph depicting the wrinkles invading the ice-solid interface upon shearing (top view and bottom view in the enlarged image). Pictures on the bottom display the resulting wrinkles on surfaces with parylene layer thicknesses of 1.2, 1.9 and 23.7 μm, respectively. (b) Contour map of the critical wave number of wrinkles as a function of the thickness of the parylene layer and the ratio of the moduli of the two layers. *E*_f_ and *E*_s_ represent the modulus of the top film and the substance, respectively. (c) A plot of the work of de-icing as a function of the parylene thickness of the peeling de-icing materials. The curves in the inset figures indicate shearing curves of de-icing. (d) Critical wave numbers and the maximum shear stress on the wrinkles as a function of the thickness of the parylene layer.

Our design strategy, illustrated in Fig. 1d, controls the surface instability between a thin, high-modulus film and a low-modulus-compliant substrate (Video S3). This instability results in a wrinkled pattern, the characteristics of which can be tailored by adjusting the modulus ratio and film thickness. Wrinkling in thin films on elastomeric substrates due to mechanical stresses is a well-studied phenomenon [29,30]. Here, we show that these surface wrinkles concentrate at the rim of the ice and weaken the interface adhesion strength (to <10 kPa), as shown in Figs 1d and 2a.

It is commonly believed that peeling stress is particularly damaging for bonded structures and significantly reduces their load-bearing capacity (Fig. S1 and Table S1) [31]. The soft substrate, due to the deformation of the material, induces the peeling effect at the interface of the cavitation [32]. Compared with soft substrates that use cavitation to weaken the ice-substrate interface, the wrinkles induced by surface instability offer a more effective method. Firstly, surface instability is universal when mechanical stress is applied to the top film, leading to immediate cavitation initiation upon the application of shear force to the ice, unlike soft substrates that require a certain degree of deformation to initiate cavitation. Secondly, the hard-modulus top surface avoids the viscoelastic properties that are typical of soft surfaces, thereby preventing the surfaces from sticking back together, i.e. Schallamach wave.

To validate the proposed design strategy, we created a model surface with a thick, soft substrate made from polydimethylsiloxane (PDMS, CA = 109.5 ± 4.5°) and a thin hard top layer from parylene (mono-chlorinated poly-*p*-xylylene [33], CA = 102.2 ± 1.4°). The high-modulus layer (parylene C) was deposited onto the PDMS film by using a parylene-deposition system. The preparation details can be found in the Methods. The modulus of the PDMS can be tuned from 0.04 to 8.63 MPa (Fig. S2). Parylene was chosen as the thin film for the model surface due to several key advantages: (i) it has a high modulus (in the range of 2.5–3.0 GPa [34]) and low surface energy, (ii) its film thickness can be precisely tuned from nanometers to micrometers and (iii) it provides strong adhesion to the PDMS substrate [35], preventing their separation during de-icing processes. Figure 1e shows the ice-adhesion strength measured from the hard, soft and TSDS (see Methods for detailed testing and set-up, and Figs S3 and S4). The TSDS (*τ* = 7.1 ± 1.8 kPa) demonstrates significantly lower ice adhesion compared with both the pure parylene surface (*τ* = 399.9 ± 75.3 kPa) and the unmodified PDMS surface (*τ* = 18.5 kPa).

### Mechanism of wrinkle-induced reduction in ice adhesion by TSDS

We first explored how the mismatched modulus impacted the ice-solid interfaces. Figure 2a depicts the state of the surface before the ice cube detaches from the TSDS. The ice-substrate contact line is encased in wrinkles, which predominantly form in front of and around the ice cube. During the act of pushing the ice cube, these wrinkles invade the ice-substrate interface, as shown in the insect of Fig. 2a. The characteristics of the wrinkles, such as their wavelength, amplitude and number, are significantly influenced by the thickness of the film (Fig. 2a bottom, and Figs S5 and S6).

It is intuitive that the greater the number of wrinkles, the more significant their influence on the initiation of cracks and thereby the breakdown of the interface. This drives us to study the characteristics of surface wrinkles. Previous research has demonstrated that the properties of wrinkles in film-substrate systems can be controlled by modifying the modulus ratio (*E*_f_*/E*_s_, where *E*_f_ and *E*_s_ represent the modulus of the hard film and the soft substrate, respectively) and the film thickness (*h*) [29,30]. The critical number of wrinkles, ${k_{\mathrm{c}}}$, can be approximated by


(2) \begin{eqnarray*}
{k_c}\,\,\sim \,\,\frac{1}{h}{\left( {\frac{{3E_s^*}}{{E_f^*}}} \right)^{\frac{1}{3}}},
\end{eqnarray*}

(3) \begin{eqnarray*}
E_s^{\mathrm{*}} = {\mathrm{\,\,}}\frac{{{E_s}}}{{( {1 - v_s^2} )}},
\end{eqnarray*}

(4) \begin{eqnarray*}
E_f^{\mathrm{*}} = {\mathrm{\,\,}}\frac{{{E_f}}}{{( {1 - v_f^2} )}},
\end{eqnarray*}

where ${E^*}$ represents the plane strain elastic modulus and ν the Poisson's ratio. Figure 2b presents a contour map showing the critical number of wrinkles as a function of the film thickness and modulus ratio (*E*_f_*/E*_s_) (Fig. S7). The parylene-PDMS system tends to have a large number of wrinkles when *E*_f_*/E*_s_ is <10^4^ and the film thickness is <10 μm, suggesting abundant cavitation initiates.

We evaluated the ice-adhesion strength by varying the number of wrinkles, which is controlled by changing the thickness of the parylene layer while keeping the modulus of the PDMS substrate constant. Figure 2c shows the work of de-icing and the ice-adhesion strength at different film thicknesses. Contrary to our previous prediction, the TSDS with a moderately mismatched modulus (e.g. parylene layer thickness of ∼6.1 μm, as shown in the inset of Fig. 2c) exhibited the lowest ice-adhesion strength, despite the fact that excessively thin films may develop numerous wrinkles. The results also suggest that thinner films require additional energy for complete ice detachment. The additional energy required could be attributed to the mode of interfacial fracture. Specifically, the de-icing curve for a TSDS with a 50-μm parylene layer shows characteristics that are akin to brittle fracture. In contrast, a TSDS with a 0.7-μm film exhibits traits that are more typical of ductile fracture—a gradual process in which plastic deformation and crack propagation occur simultaneously, continuously consuming a significant amount of energy [36].

To clarify the impact of wrinkles on adhesion strength during the de-icing process, we used a finite-element (FE) model to simulate the stress distribution within the wrinkles across different film thicknesses. We initially varied the film thickness from 1.8 to 24 μm and the resulting wrinkle formation closely matched that of the experimental data, as shown in Fig. 2d. Wrinkle count and the simulated peak stress distribution are plotted against the film thickness. Notably, as the film thickness increases, the stress concentration rises, showing a trend that is opposite to that of wrinkle formation. Moreover, the underlying mechanism of wrinkles formation is not affected by ice-cube size or shape. It should be noted that the out-of-plane amplitude of the wrinkles also plays a key role in initiating cracks at the ice-substrate interface. The interface between the ice and substrate, where wrinkles meet the flat ice surface, is particularly vulnerable to disruption. Larger out-of-plane wrinkles are more likely to cause tearing at the ice-substrate interface. In essence, while thinner films produce more wrinkles, the smaller out-of-plane amplitude and reduced stress concentration in these wrinkles are insufficient to disrupt the ice-substrate interface (see Fig. 2c and Fig. S8). Additionally, the ice adhesion could be further reduced if the active method is adopted to generate the wrinkles (Fig. S9).

### The synergetic effect of wrinkles at two length scales on the interfacial crack boosting

**Figure 3.**
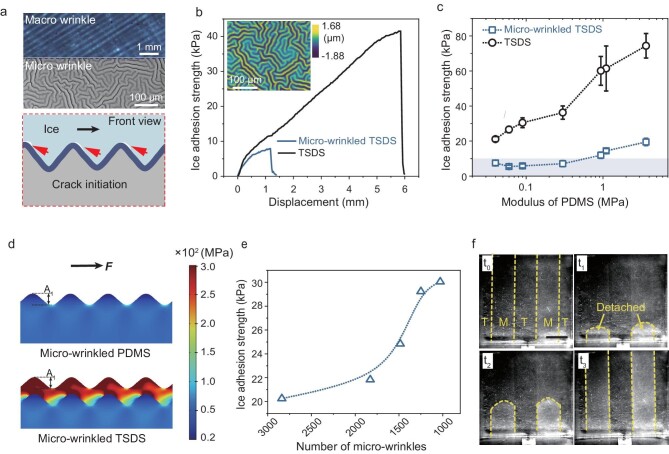
Optimization of designing micro-wrinkled TSDSs. (a) Schematic showing the synergetic effect of macro- and micro-wrinkles on the ice adhesion. (b) De-icing curves of the TSDS and micro-wrinkled TSDS, respectively. Insert is the image of the Turing micro-wrinkles taken from white-light interference microscopy. (c) Ice-adhesion strength as a function of the modulus of PDMS (substrate) for the two surfaces. (d) Photos of the finite-element models displaying stress distribution upon shearing on the two types of surfaces. The top is the wrinkled PDMS surface with an amplitude of 2 μm and the bottom is the micro-wrinkled TSDS with the same amplitude. (e) A plot of the ice-adhesion strength as a function of the number of micro-wrinkles per area. (f) Surface with a striped pattern composed of TSDS (T) and micro-wrinkled TSDS (M) regions. Micro-wrinkled TSDS regions are detached earlier than TSDS regions. The area with a dotted line indicates the detached interface.

The measurement of ice adhesion on the TSDS suggests that surface instability-induced wrinkles significantly impact ice adhesion. This leads us to consider whether pre-generated wrinkles could further disrupt the interface and initiate cracks (Fig. 3a). To test this hypothesis, we prepared TSDS with pre-generated micro-wrinkles (termed as micro-wrinkled TSDS in the following discussion). We employed the wrinkles with a Turing pattern that was generated during the deposition process of the parylene film, as detailed in Fig. S10 (refer to Methods for fabrication specifics). Wrinkles with a Turing pattern are well documented in thin-film preparation through surface instability [37–41]. As shown in the insert of Fig. 3b, the isotropic Turing pattern was uniformly distributed on the surface. It should be noted that the Turing pattern was employed due to its isotropy, which eliminates the influence of the applied stress direction on the adhesion strength (Fig. S11) [42]. The characteristics of this pattern can be easily modified by the moduli of the PDMS, as illustrated in Figs S12 and S13. Compared with macro-wrinkles that are generated by shear stress, those with dual-scale peeling sites exhibit a significant reduction in ice-adhesion strength, dropping to <10 kPa (Fig. 3b and c, and Video S4).

It is commonly believed that the introduction of roughness, such as wrinkles, would increase ice adhesion due to the increased ice-substrate contact area. However, it is surprising to observe that wrinkles significantly decrease ice adhesion in the micro-wrinkled TSDS system. To understand the mechanism behind this, we developed an FE model to simulate the stress distribution on surfaces with and without micro-wrinkles under shearing conditions. Figure 3d illustrates the comparison of stress distributions, showing a significantly high stress concentration on the micro-wrinkled TSDS. On the other hand, micro-wrinkled soft surfaces exhibit almost no stress concentration under shearing conditions. The concentration of the shear stress is attributed to the synergistic relationship between the micro-wrinkle and the mismatched modulus. To validate these findings, we measured the ice adhesion on constant- and mismatch-modulus substrate with the same micro-wrinkle to elucidate the role of the micro-wrinkle and mismatched modulus, as detailed in the Methods (Figs S15 and S16). Fig. S17 shows that a significant reduction in ice-adhesion strength is only achieved when both micro-wrinkles and modulus mismatches are present. Conversely, the presence of micro-wrinkles alone not only fails to reduce adhesion strength, but may even increase it. This clearly demonstrates the synergistic effect between micro-wrinkles and mismatched-modulus macrostructures. We then investigate the impact of the wrinkles size (number) on the ice-adhesion strength (Fig. 3e). Different numbers of micro-wrinkled samples are fabricated via a molding process (Fig. S15). For a smaller size, the stress concentration is higher, resulting in a smaller ice adhesion. To make the contrast more obvious, we prepared a composite surface that consisted of striped TSDS (T) and micro-wrinkled TSDS (M) regions (Fig. S18; see Methods for details on fabrication). Video S5 and Fig. 3f indicate that the micro-wrinkled TSDS regions are detached first, even though they are surrounded by TSDS, which means micro-wrinkled TSDS regions are unable to generate macro-wrinkles on either side. On the contrary, no interface fracture has been observed in the TSDS, and even complete separation was completed in the micro-wrinkled TSDS region.

### Applicability and application of micro-wrinkled TSDSs

**Figure 4.**
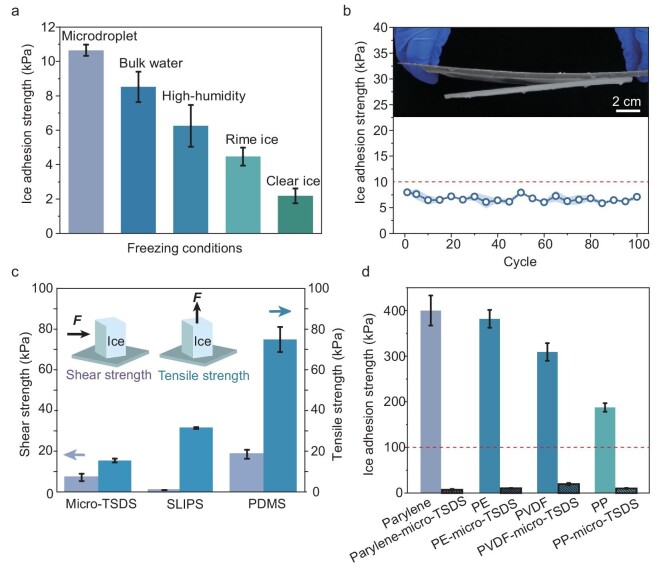
Applicability and application of micro-wrinkled TSDSs. (a) Ice-adhesion strength measured for micro-wrinkled TSDSs under different freezing conditions. (b) Influence of icing and de-icing cycles on de-icing performance. Inset shows the shedding of ice by gravity. (c) Shear strength and tensile strength of three typical surfaces made of micro-TSDS, SLIPS and PDMS, respectively. (d) Adaptability of the TSDS strategy on different engineering materials.

As de-icing materials are commonly used in the real world for complex freezing conditions, we tested the de-icing properties of micro-wrinkled TSDSs under common icing conditions, including micro-droplets, bulk water, high humidity, rime ice and clear ice (Fig. 4a). ‘Bulk water’ means adding water to the surface and freezing it *in situ*, which is a static freezing method. The icing methods that are not specified in the article are all performed in this way. The ‘high humidity’ is a condition with ∼90% relative humidity (Figs S19 and S20; see Methods for details on the experiment). The ‘rime ice’ and ‘clear ice’ icing conditions are achieved in an icing wind tunnel (see Methods for details on the experiment, Fig. S21 and Table S2). The results display that micro-wrinkled TSDSs show a great de-icing effect (*τ*_ice_ < 10 kPa) under the mentioned conditions. Furthermore, when the icing temperature is varied from –10°C to –50°C, there is a limited effect on the adhesion strength of the micro-wrinkled TSDSs (Fig. S22). These results indicate the micro-wrinkled TSDSs have excellent performance stability. The mechanical stability was further examined by using a series of tests. Figure 4b shows that the micro-wrinkled TSDS maintained an ice-adhesion strength of <10 kPa even after 100 icing-de-icing cycles and it is supposed that the high-modulus top layer performs as a robust crust to protect the soft inside. It also can be proved by repeating the tape-peeling test >500 times (Fig. S23). In addition, other severe durability tests were conducted, including ultraviolet aging (Fig. S24), resistance to chemical corrosion (salt spray atmosphere with 5 wt% of NaCl solution for 31 days, Fig. S25) and the antifouling test (Fig. S26). Our micro-wrinkled TSDSs maintain great de-icing properties under these conditions, which indicates that the micro-wrinkled TSDSs have the potential to be applied in harsh environments.

Considering the practical application conditions of de-icing materials, not all cases are achieved by applying shear forces for de-icing. The ice accretion should be able to detach spontaneously from the desired de-icing materials through natural forces (gravity, wind) or as little energy as possible. Both the shear strength and the tensile strength of different types of low-adhesion-strength surfaces have been measured (Fig. S27), including those of the micro-wrinkled TSDS, slippery substrate (SLIPS) and soft substrate. As shown in Fig. 4c, the tensile strength of all the material systems shows an increase compared with the shear strength. However, the micro-wrinkled TSDS expresses the smallest difference between these two types of strength due to the same mechanism of micro-/macro-wrinkles. A slippery surface provides a small shear strength due to the oil at the interface between the ice and the substrate, although the capillary force of the oil makes the tensile strength high. As for the soft substrate, the same trend was observed with a tensile strength that was much higher than the shear strength. This is majorly attributed to less crack initiation by the tensile stress at the interface. On the contrary, the micro-wrinkled TSDS has a relatively low tensile strength (15.4 ± 0.9 kPa).

To verify the universality of the mismatched-modulus strategy, we demonstrated the reduction of ice adhesion with multiple types of materials. Different kinds of micro-wrinkled TSDSs were fabricated, including PVDF (polyvinylidene difluoride)-micro-wrinkled TSDS (elastic modulus of PVDF is 2.5 GPa [43]), PE (polyethylene)-micro-wrinkled TSDS (elastic modulus of PP is ∼1 GPa [44]), PP (polypropylene)-micro-wrinkled TSDS (elastic modulus of PP is 1.14–1.55 GPa [45]), by using a hot press (Fig. S16; see Methods for details). The fabrication of micro-wrinkles on surfaces is well established, including methods such as thermal instability, hot pressing, photoinduction and solvent induction [29,30,37,38,46,47], many of which are suitable for large-scale production. Figure 4d shows the measured ice-adhesion strengths for several high-modulus materials and the corresponding micro-wrinkled TSDSs. These materials all achieve the effect of efficiently reducing ice-adhesion strength through the design principle of micro-wrinkled TSDS.

To evaluate the application potential of micro-wrinkled TSDSs, we investigated ice shedding by centrifugal force on a simulated wind turbine (Figs S28–S30 and Video S6; see Methods for details on surface preparation). Ice on the micro-wrinkled TSDSs detached automatically at relatively low centrifuge velocities, with line speeds of <30 m/s, while ice remained adhered to other surfaces, such as superhydrophobic and metal surfaces, even at 40 m/s. Additionally, self-shedding of ice under gravity was demonstrated (inset of Fig. 4b, Fig. S31 and Video S7; see Supplementary Methods for details). A 1 m × 0.7 m micro-wrinkled TSDS was fabricated and fixed to a larger aluminum sheet, allowing a 0.9 m × 0.6 m ice layer to form at –10°C in a walk-in freezer. Once fully frozen, the ice detached completely when the aluminum plate was tilted upright (Video S8; see Supplementary Methods for details). The spontaneous shedding of ice of various thicknesses suggests that the micro-wrinkled TSDS surface may facilitate automatic de-icing through natural forces.

To summarize, the micro-TSDSs demonstrate excellent icephobic properties and durability under laboratory conditions, as well as in preliminary tests. However, given the complexity of real-world freezing conditions and the need for efficient de-icing, it is important to explore the combination of the micro-TSDS with an active anti-icing system, which may amplify the benefits of both low adhesion and rapid crack propagation, enabling more effective ice removal.

## CONCLUSIONS

In this study, we propose a strategy to design de-icing surfaces by generating dual-scale crack initiates at/in the ice-solid interface. The designed micro-wrinkled TSDS displays excellent de-icing performance, taking advantage of surface instability-induced wrinkles. Following the design principle, we verified the universal applicability of this design strategy and its effectiveness in different environments. The protective skin significantly enhances the robustness of the TSDS, as demonstrated through a series of environmental tests. We envision that this designed surface is expected to have broad significance for the de-icing field, especially in complex and harsh icing environments.

## METHODS

### Materials

PDMS (Sylgard 184, Dow Corning), parylene C (DPX-C, dichloro-*p*-cyclophame), trichloro (1H,1H,2H,2H-perfluorooctyl) silane (Sigma-Aldrich, 97%), hydrogen fluoride (Aladdin, 49%), ammonium fluoride (NH_4_F) (Aladdin, 98%), tetramethylammonium hydroxide (TMAH, 25% in water) and test dust (ISO 12103–1, A2 FINE) were used as received.

Silicon oil, PMX-200 (viscosity ∼1000 mPa·s), was purchased from Aladdin. Polyethylene (PE) film, polypropylene (PP) film and polyvinylidene difluoride (PVDF) film were purchased from KeXin Electronic Material Co., Ltd. The silicon wafer (100) with a 300-nm-thick oxide layer was purchased from Suzhou Research Materials Microtech Co., Ltd.

### TSDSs

To fabricate the TSDSs, PDMS (the mixture of the Sylgard 184 elastomer kit and curing agent) was used to create the low-modulus layer and was designed with 50:1 cross-linker ratios. The mixture was stirred until homogeneous, degassed to remove all bubbles then carefully poured into a mold, which consisted of an aluminum bottom and a 2-mm-thick rubber pad, which featured a precisely designed hollow shape at its center, which was intended to define the final geometry of the cast material. After all bubbles had dissipated, the entire mold was placed in an oven at 80°C for hours to complete the curing process. Upon curing, the rubber mold was removed and the prepared PDMS film was subjected to plasma treatment for 1 minute in an air atmosphere. The high-modulus layer (parylene C) was evaporated onto the PDMS film by using a parylene-deposition system. Specifically, the parylene film was fabricated by using the SCS (Special Coating Systems) vapor deposition system, allowing precise control over the coating rate and thickness. In the deposition process, the powdered precursor (dimer) was vaporized under a vacuum and heated to form a dimeric gas. The gas was then pyrolysed to cleave the dimer into its monomeric form, which was subsequently deposited as a transparent polymer film. Parylene coatings can be applied in thicknesses that range from several hundred angstroms to 75 μm. The thickness of the parylene layer could be regulated by the addition mass of the parylene (Fig. S5).

### Micro-wrinkled tough-skin de-icing surfaces (micro-wrinkled TSDSs)

In the parylene-deposition process, the powdered precursor (dimer) was vaporized under a vacuum and heated to form a dimeric gas. This gas was then pyrolysed to cleave the dimer into its monomeric form, which was subsequently deposited as a transparent polymer film on the PDMS. At the early stages of the chemical vapor deposition (CVD), the porous nature of PDMS allows the parylene monomer to diffuse into the PDMS polymer during deposition. The modulus mismatch between parylene and PDMS induces wrinkle formation.

### Shear adhesion measurement

As Fig. S3 shows, a homemade test system was employed to test the shear adhesion strength. The refrigeration system consisted of a customized cooling platform and a cooling cycle (cooling liquid: a mixture of ethylene glycol and water). Distilled water was poured into the handmade acrylic cuvette, then the ice was frozen *in situ* at –20°C. The 3D sizes of the ice were fixed at 2 × 2 × 0.5 cm (length × width × height) and the sample with a metal plate was adhered to the cooling platform via ice. Once frozen fully, the force gauge (Mark 10) was used to detach the ice at a constant velocity of 0.5 mm/s. The maximum force (*F*) needed to dislodge the ice was recorded and the reported values were the average of a minimum of three measurements.

### FE modeling of TSDS

In this work, ANSYS and COMSOL were used to conduct the FE modeling. In detail, the in-plane force-induced deformation and stress change with 3D vision was performed on ANSYS, while the cross-plane stress distribution of the micro-wrinkled PDMS and micro-wrinkled TSDS base in the de-icing situation was simulated by using COMSOL software. Details of the simulation setting can be found in the supporting note.

## Supplementary Material

nwaf005_Supplemental_Files
