# Supplementary material for: A skin-inspired durable de-icing surface with boosting interfacial cracks
Source: Natl Sci Rev. 2025 Jan 13;12(3):nwaf005. doi: 10.1093/nsr/nwaf005 (PMC11827584; doi:10.1093/nsr/nwaf005)
Supplement: nwaf005_Supplemental_Files [file nwaf005_supplemental_files.zip › Supplementary data.pdf]

Supporting information

A skin-inspired durable de-icing surface with boosting interfacial cracks

Qiucheng Yang[1], Jinlong Yang[1*], Yuhao Hu[1,] Xiaopeng Niu[2], Zhenda Liu[1], Jian Zou[3], Junchang Guo[1], Hao Xiong[4], Xingshi Gu[4], Li Yang[5], Fanfei Yu[6], Shunpeng Zhu[2], Ming Ye[7], Xian Yi[4], Xu Deng[1,8*]

Supplementary Videos

Video S1. Comparing shear force and peel force. Ice cube was removed from PVDF film with 30 μm by shearing/peeling. The ice freezing at -20 ℃, the size of the ice is 2 × 2 × 1 cm^3. The speed of the putter is 0.5 mm/s.

Video S2. The Schallamach waves associated with stick-slip movement for ice shearing on low-modulus surfaces. Shearing the ice from a low-modulus PDMS surface and a TSDS. For PDMS, trapped air cavities propagate as pulses along the ice-solid interface during shearing. The detached interface re-attaches, causing the ice to slip continuously along the surface. On the TSDS, there is no stick-slip movement but some macro-wrinkles around the ice. The ice freezing at -20 ℃, the size of the ice is 2 × 2 × 0.5 cm^3. The speed of the putter is 0.5 mm/s.

Video S3. Shearing ice from the TSDS. The process of removing ice from a TSDS surface by shearing, and a corresponding shear curve. The stress-induced macro-wrinkles are obvious in this process. The ice freezing at -20 ℃, the size of the ice is 2× 2 × 0.5 cm^3. The speed of the putter is 0.5 mm/s.

Video S4. Shearing ice from the micro-wrinkled TSDS. The process of removing ice from a micro-wrinkled TSDS surface by shearing, and a corresponding shear curve. The formed macro-wrinkles invade the ice-solid interface easily. The ice

adhesion strength reduces sharply than it of the TSDS. The ice freezing at -20 ℃, the size of the ice is 2 × 2 × 0.5 cm^3. The speed of the putter is 0.5 mm/s.

Video S5. Shearing ice from the striped pattern surface. Removing ice from the striped pattern composite surface consisted of striped TSDS (T) and micro-wrinkled TSDS (M) regions. The ice freezing at -20 ℃, the size of the ice is 4 × 4 × 0.5 cm^3. The speed of the putter is 0.5 mm/s.

Video S6. Ice shedding on simulated blade of wind turbine. The blades of simulated wind turbine were covered by SHS surface, metal surface, and micro-wrinkled TSDS surface, respectively. Ice was formed on these surfaces at -10 °C in a walk-in freezer overnight. The process was recorded by a high-speed camera.

Video S7. Self-shedding of ice under gravity. Shedding the ice by gravity from the micro-wrinkled TSDS and PDMS surface, respectively. The ice freezing at -20 ℃, the size of the ice is 19 × 19 × 1.0 cm^3.

Video S8. Large area de-icing. A 1 × 0.7 m micro-wrinkled TSDS was fabricated and fixed to a larger aluminum sheet, allowing a 0.9 × 0.6 m ice layer to form at -10 °C in a walk-in freezer. After freezing (overnight), the ice detached completely when the aluminum plate tilted upright.

Supporting note

Fabrication of surfaces

TSDS with striped pattern. As described above, the prepared PDMS was then masked with a blue film with striped holes, in which the width of the holes and the spacing between the holes are both 1 cm. The blue-film-covered PDMS was treated with plasma for 1 min (air atmosphere). After that, the blue film was removed, and the parylene C film was evaporated onto the PDMS substrate to obtain the stripped pattern PDMS (Figure S18).

TSDS with the micro-striped pattern. The combination of photolithography and complex method prepared the parylene-TSDS with the micro-striped pattern (Figure S11). The micro-striped pattern was prepared on silicon substrates by wet etching. Briefly, the commercial SiO_2/Si wafer was patterned using photolithography in a square lattice, and the patterned wafer with photoresist was etched by BOE (Buffer-Oxide-Etch, 40 % NH_4F:49 % HF = 6:1) solution to remove the open oxide layer. The photoresist layer was removed through immersion in boiling acetone, and The Si substrate was subsequently etched by using the TMAH solution (25 %) at 70 °C. Finally, the oxide mask was removed by treatment with BOE solution. The dimensions and features of the resulting microstructures were precisely defined by the designed photomask.

The fabricated Si wafer with microstructures was treated by plasma for 5 min and subsequently chemically functionalized with trichloro (1H,1H,2H,2H-perfluorooctyl)

silane by CVD. Fluoridization treatment was for the purpose of easy demolding. The degassed prepolymer PDMS (10:1 cross-linker ratio) was poured into and cured. Finally, the sample was treated with plasma and the parylene film was evaporated onto the PDMS by a parylene deposition system.

Low-modulus surface with micro-wrinkles. The Turing microstructure of the micro-wrinkled TSDS is defined as a mold-1 (Figure S15). It was treated by plasma for 5 min and then Fluoridization. The prepolymer of PDMS was poured into the mold and cured at 80 °C for 2 h. The PDMS mold-2 with the inverted micro-structures was obtained after peeling off from the mold-1. This inverted mold-1 was fluoridized as mentioned, and the degassed PDMS precursor was poured into it and cured at 80 °C for 2 h. After peeling the inverted mold-2, the target sample was obtained.

High-modulus surface with micro-wrinkles. The microstructure of micro-wrinkled TSDS is defined as a primary mold-1 (Figure S15). As mentioned, a PDMS (10:1 cross-linker ratio) mold-2 with an inverted microstructure was obtained. Using the PDMS mold-2 as a template, a nanoimprint machine prepared PMMA (polymethacrylates) with positive microstructure. The set temperature (95 °C) was according to the melting point of PMMA. Then, the parylene was spurted onto the micro-wrinkled PMMA substrate.

TSDS with different numbers of micro-wrinkles. To eliminate the effects of modulus and film thickness variations. Different numbers of micro-wrinkled samples are fabricated via a molding process (Figure S15). The size of the microwrinkles is primarily determined by adjusting the modulus of the PDMS during the molding process. To ensure a constant modulus for the final samples, we used PDMS with a 10:1 curing ratio, which minimizes modulus variability. After molding, these micro-PDMS samples were coated with Parylene C (using the same feed to maintain consistent thickness) following a 1-minute plasma treatment. This process results in micro-TSDS with different sizes of microwrinkles

PE/PP/PVDF micro-wrinkled TSDSs. PE micro-wrinkled TSDS, PP micro-wrinkled TSDS, and PVDF micro-wrinkled TSDS were fabricated by heat press. As above, a PDMS (cross-linker ratio = 10:1) mold-2 with an inverted microstructure was obtained. The mold-2 was used to prepare PMMA (polymethacrylates) with positive microstructure (mold-3) by nanoimprinting. The set temperature (95 °C) was according to the melting point of PMMA. The inverted PMMA mold-4 was similarly prepared by nanoimprinting. Then, the desired micro-wrinkled PE film (10 μm), PP film (10 μm), and PVDF film (10, 30 μm), were prepared by heat press (temperature is according to their melting points). The degassed PDMS precursor was poured onto the back of the micro-wrinkled film. After curing, the target sample was fabricated, and the schematic diagram of the process was shown in Figure S16.

Slippery surface. The PDMS film (2-mm thickness, 10:1 cross-linker ratios) was immersed in silicon oil PMX-200 for one week. The slippery surface was used in the self-cleaning test, compared with micro-wrinkled TSDS surface (Figure S26).

Superhydrophobic surface (SHS). The SHS surfaces were prepared by spraying the Glaco onto the aluminum sheet.

Ice adhesion Characterization

Tensile adhesion measurement. Based on the shear ice adhesion test platform, a simple transformation was carried out (Figure S27). Change the mobile platform from the horizontal to the vertical direction. The ice mold was customized with a hook and a water injection hole on the top. The size of the ice was fixed at 2 × 2 × 1.5 cm. Other testing conditions are the same as the shear adhesion test.

Gravity self-shedding. Micro-wrinkled TSDS (20 × 20 × 0.2 cm) and PDMS surfaces (50:1 cross-linker ratio) were prepared with a metal bottom. The homemade cuvette (rubber, 19 × 19 × 0.5/0.4/0.2 cm) was added to water and re removed carefully after freezing. The ice was freezing in situ at -20 °C. These ice-covered samples were overturned to verify self-shedding effect. These processes were recorded by a digital camera (Figure S31, Video S7).

Large area de-icing demonstration. A 1 × 0.7 m micro-wrinkled TSDS, fixed on a larger aluminum sheet, was prepared. A prepared 0.9 × 0.6 m PMMA mold was used to form ice on the surface at -10 °C overnight, in a walk-in freezer. After fully frozen, the sample attached ice was placed vertically, and then, the large area of ice could be

spontaneously and completely detached from the micro-wrinkled TSDS. The whole process was recorded by a digital camera (Video S8).

Simulation of wind turbine blade. A pint-sized wind turbine blade was prepared by customized small rotor and some corresponding single-detachable aluminum blades. A high-speed agitator with adjustable speed was modified to act as an engine for the rotor blade (Figure S29). We prepared 4 kinds of surfaces (parylene micro-wrinkled TSDS, PE micro-wrinkled TSDS, PVDF micro-wrinkled TSDS, and SHS) on the customized aluminum blades for this test, and their shear adhesion strengths were displayed in Figure S28. We moved this assembled setup into a walk-in freezer at -5 to -7 °C to simulate the cold environment. The rubber cuvette was used as the mold for ice, and it was removed carefully after freezing. The ice was freezing in situ in the walk-in freezer overnight with fixed size (9 × 3.5 × 3 cm) After that, these ice-covered blades were fixed on the rotor blade quickly. Then, the setup started to spin at the set speed (Figure S30, Video S6). These spinning processes were recorded by a high-speed camera and were analyzed using Photron FASTCAM Viewer 4.

Surface characterization. Surface morphology was observed using scanning electron micrography (SEM, Phenom Pro X), white-light interference microscopy (Sensofar, S Neox 090), and optical microscopic. The static water contact angle measurement was conducted using an OCA 50 AF (Dataphysics). A sessile drop (5 μL) was dispensed onto the test surface with a pipette. The side-view image was captured when the droplet was stabilized. An average of five different locations was reported. The thickness of the parylene layer (parylene was sputtering onto a silicon wafer) was

measured using the thickness gauge (Filmetrics). The average of three different locations was reported. Moduli of samples were tested by nanoindentation.

Performance characterization

Different freezing conditions. In this work, statistic freezing condition means freezing in situ: adding untreated water (tap water) into the mold which was put on the sample to the desired height and, freezing in situ at the target temperature. Here, dynamic freezing conditions include high humidity, micro-droplets, rime ice, and clear ice. The high-humidity environment was achieved by increasing the humidity inside the testing chamber with a humidifier. Maintain humidity at 93 % for 8 h (Figure S19-20). The micro-droplet condition means the mist is sprayed directly onto the surface. The testing of rime ice and clear ice was completed in an icing wind tunnel. The specific test conditions are shown in Supplementary Table S2 (Figure S21).

Freezing temperature. To show the stability of de-icing properties of the micro-wrinkled TSDS in different freezing conditions with various temperatures (range from -10 to -50 °C).

Icing/Deicing cycle. We selected the micro-wrinkled TSDSs to test their durability after icing/deicing 100 cycles. The temperature for freezing is -20 °C and the size of ice is 4 cm^2.

Self-cleaning test. Impurities and dust are inevitable in the natural environment. In this test, we compared the parylene micro-wrinkled TSDS and the slippery (silicon

oil-PDMS) surface. The dust particles were covered on these 2 surfaces and then washed with water. On the parylene micro-wrinkled TSDS the dust particles were grabbed away by water, leaving behind a clean surface. While the slippery one still has some dust particles accumulated on the surface (Figure S26).

Mechanical and Chemical characterization

Tape-peeling test. The tape-peeling tests were conducted to evaluate the mechanical stability of the parylene micro-wrinkled TSDSs. The 3M™ VHB™ 5925 tape (with adhesion to steel value of 3000 N m-1) was employed for the tape-peeling test.

Salt spray test. When de-icing materials are used in certain environments, such as base ships, salt spray corrosion resistance is one of the essential properties of deicing materials. The slat spray tests were conducted to evaluate the stability of the parylene micro-wrinkled TSDS, PVDF micro-wrinkled TSDS, and PE micro-wrinkled TSDS. These 3 types of micro-wrinkled TSDSs were put in a salt spray testing machine for 10 days, these samples were taken out and clean to test the ice adhesion strength. After that, samples were put back into the salt spray testing machine again. The concentration of salty water is 5 wt%.

Ultraviolet aging test. UV resistance presents a significant challenge that must be addressed when employing de-icing materials in real-world applications, particularly in high-altitude environments. The PVDF micro-wrinkled TSDS sample was placed in a UV aging test chamber for 10-day, and its de-icing adhesion strength was subsequently evaluated.

Simulation of the shear stress distribution on the macro wrinkled. According to the buckling theory, when the applied load on a structure reaches a certain value, a small increment in the load can result in a significant change in the equilibrium configuration of the structure. Thin membrane structures develop wrinkles under external loads, subsequently affecting mechanical performance and deformation. This paper utilizes thin shell elements to simulate the wrinkling phenomenon in membrane structures under in-plane loads, obtaining information such as wrinkle wavelength, amplitude, and stress-strain characteristics. Specifically, an explicit dynamic analysis approach is employed, using the ANSYS/LS-DYNA shell element to conduct nonlinear buckling analysis on the forming of wrinkles in membrane materials. Since nonlinear buckling analysis requires the structure to be "imperfect", meaning it requires an initial perturbation or defect, eigenvalue buckling analysis can predict the approximate location of critical instability forces. Therefore, for nonlinear buckling analysis, the initial perturbation is based on eigenvalue buckling analysis. In ANSYS APDL 17.0, the shell 181 element is used to establish a finite element model, set boundary conditions, apply pre-stress, and perform eigenvalue buckling calculations to obtain the buckling modes. This process generates a finite element model with initial imperfections, taking the deformation results of the first few modes and applying a scaling factor as a small initial perturbation. Subsequently, the arc-length method is employed to perform nonlinear buckling analysis, obtaining the final form of wrinkles and stress-strain responses.

Simulation of the stress distribution in micro-wrinkled TSDS with compression. Micro-wrinkled TSDS system is a typical multi-body system coupled with in-plane force. When moving the accumulated ice on the (micro-wrinkled/flat surfaces) hard parylene layer, the shear force between ice/surface contact couples leads to a stress increase and deformation. To evaluate the influence of surface morphology and the inner stress distribution, COMSOL software was applied to simulate the ice-pushing process. In the modeling, a parameterized curve was used to simulate surface wrinkling. In the wrinkled case, a sine wave with a period of 15 μm and an amplitude of 2 μm was used, while the thickness of parylene was set at 6 μm. The material parameters of parylene can be acquired in ref[4], and the ice parameters can be found in the embedded material library of COMSOL. In the initial physical field configuration, the bottom of micro-wrinkled TSDS was fixed and the left/right side of micro-wrinkled TSDS and parylene was free. A fixed offset of one wave period is applied on ice to simulate its movement. To get a high-resolution interface stress distribution image, an extremely fine grid was applied on the key area such as the whole parylene and the near surface of micro-wrinkled TSDS.

Supplementary Figures

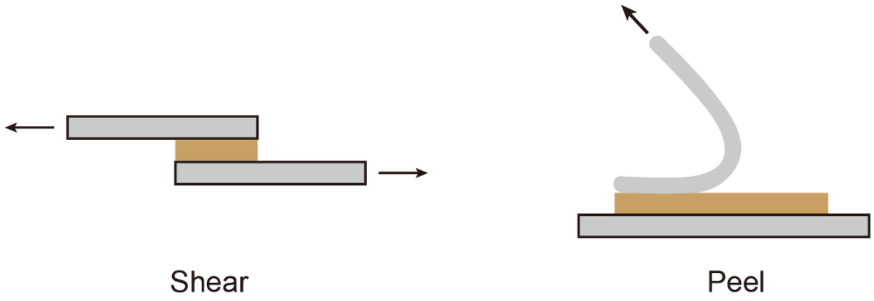

Figure S1. Schematic of shearing stress and peeling stress.

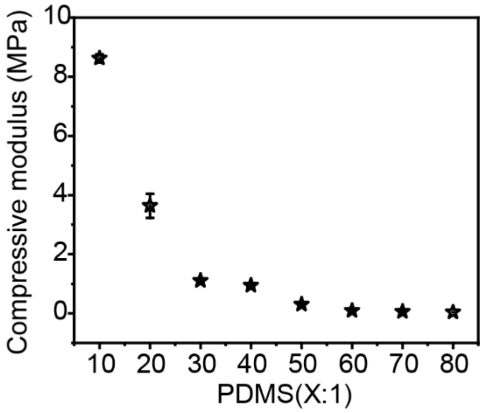

Figure S2. Compressive modulus of PDMS against cross-linker ratio (X:1). The “X” means the proportion of prepolymers. The data were obtained by a universal test machine.

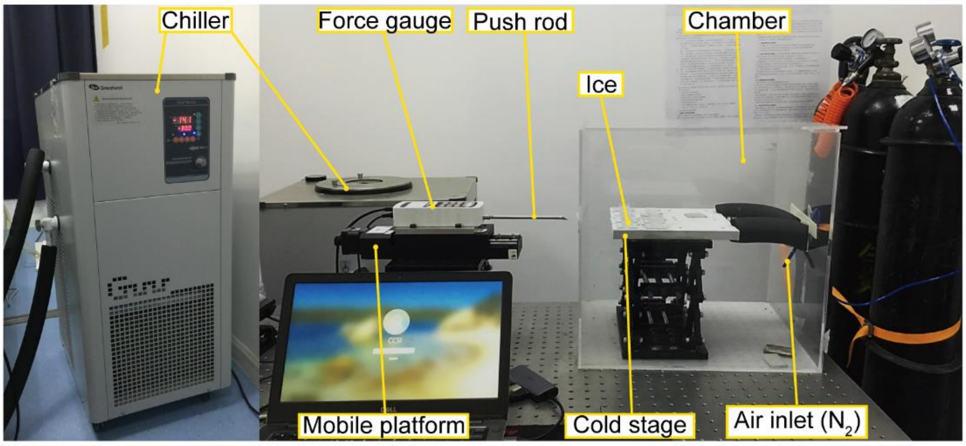

Figure S3. The homemade shear adhesion test platform.

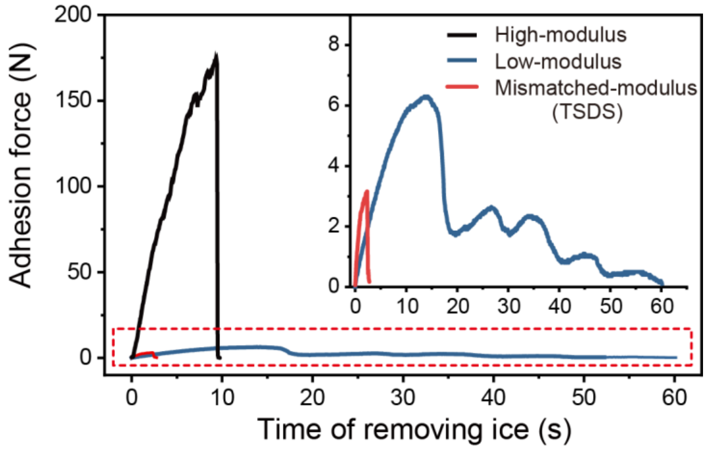

Figure S4. Force as a function as the time of removing ice on three kinds of surfaces.

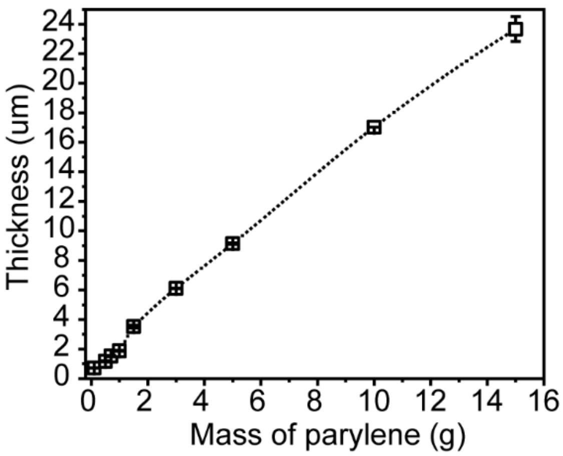

Figure S5. The thickness of the parylene layer as a function of the amount of feedstock.

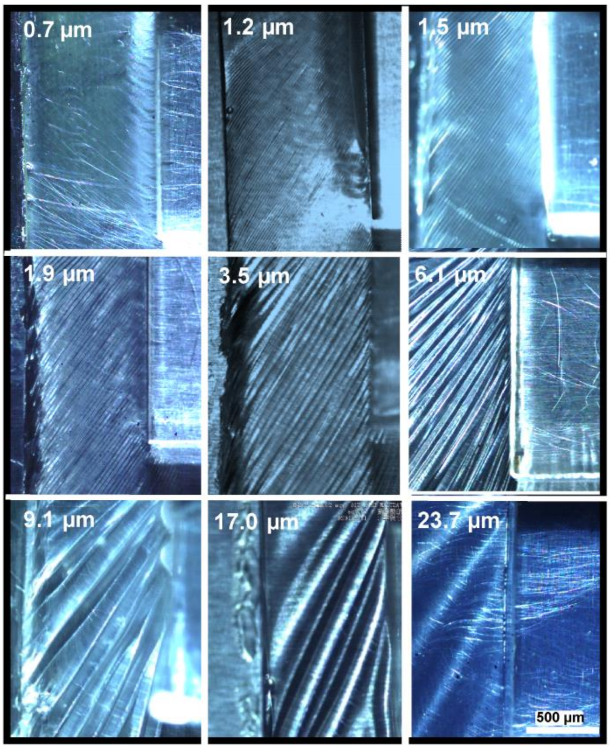

Figure S6. TSDS with various parylene thicknesses. The macro-wrinkles form at the rim of the cube which is applied by shearing force.

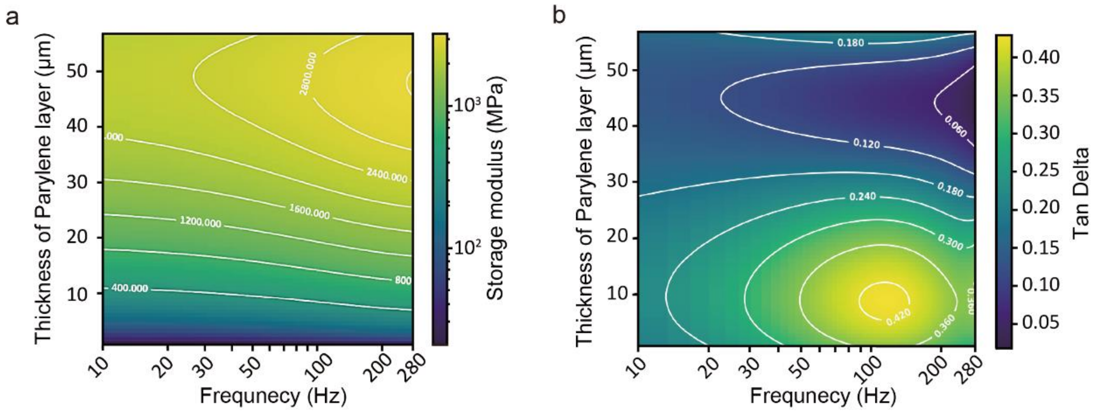

Figure S7. The modulus information of the parylene-TSDS surface was obtained by nanoindentation test**. a,** Storage modulus of the whole parylene-TSDS as a function of the thickness of the parylene layer and the frequency. **b,** Tan delta as a function of the thickness of the parylene layer and the frequency.

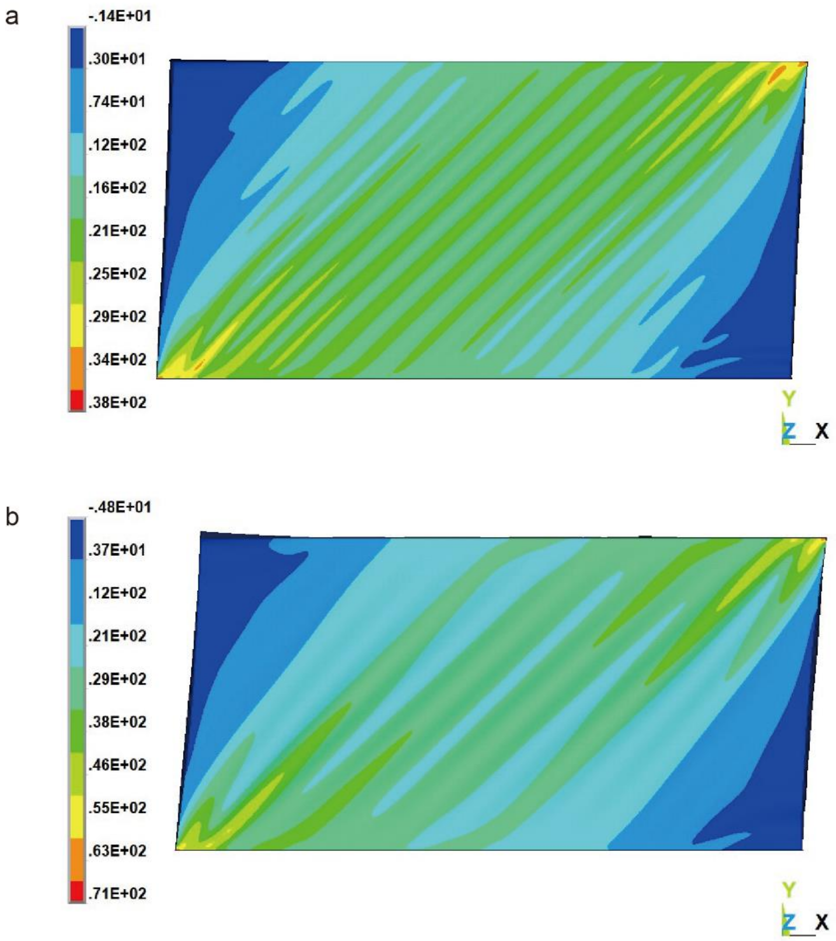

Figure S8. The pictures of the finite-element model. position the surface with varying parylene thickness to achieve uniform stress distribution in the XY direction. a, parylene film thickness = 6.12 μm; b, parylene film thickness = 23.7 μm

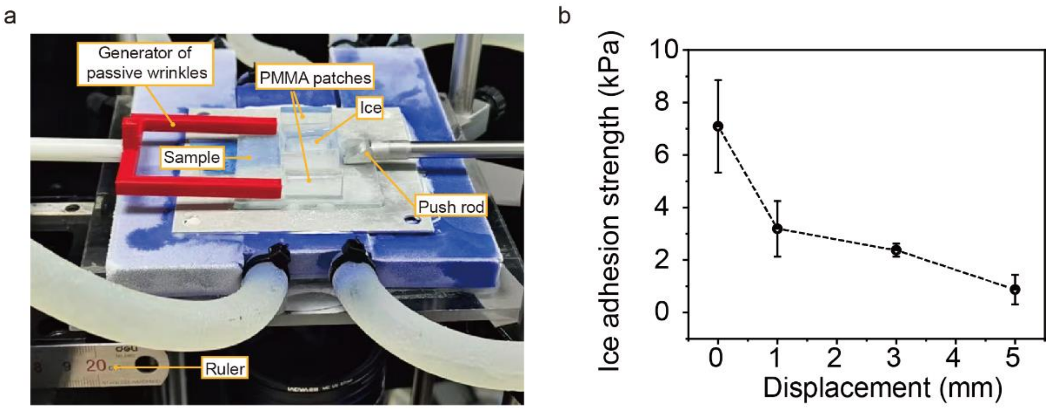

Figure S9. a. The setup to generate passive wrinkles around the ice, then sheared the ice off to measure the adhesion force. The degree of passive wrinkle is depended to the displacement of the "generator of passive wrinkles"; b. Ice adhesion strength as a function of the degree of passive wrinkles (displacement)

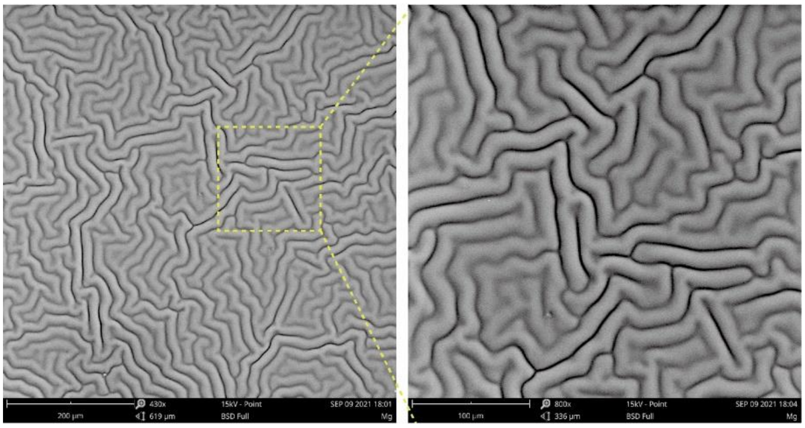

Figure S10. Scanning electron microscopy (SEM) image of micro-wrinkled TSDS which is fabricated by vacuum sputtering. The thickness of the parylene layer is 3.5μm, and the PDMS with a cross-linker ratio (50:1).

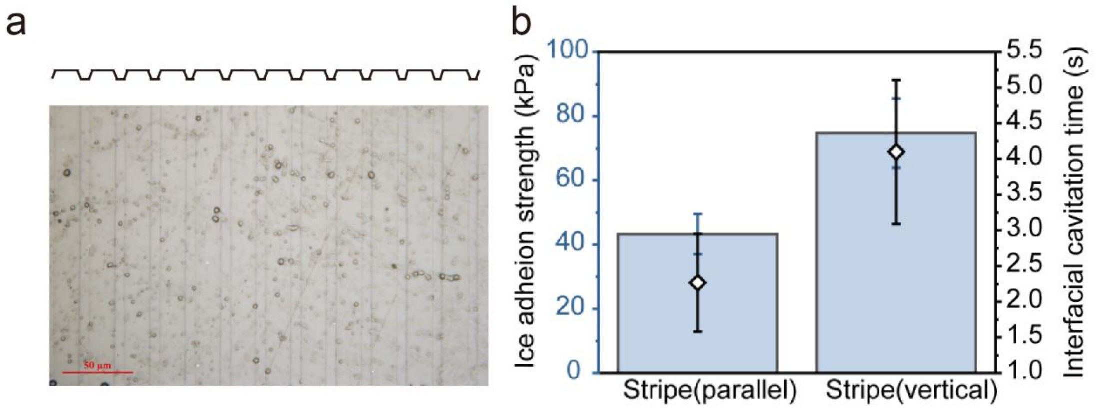

Figure S11. a, A composite of micro-striped TSDS((parylene(3.5 μm)-PDMS(10:1)). The PDMS with a cross-linker ratio (10:1). **b,** Shear force is applied parallelly or vertically to the direction of the micro-stripe. The corresponding shear adhesion strength and the time required to detach the ice.

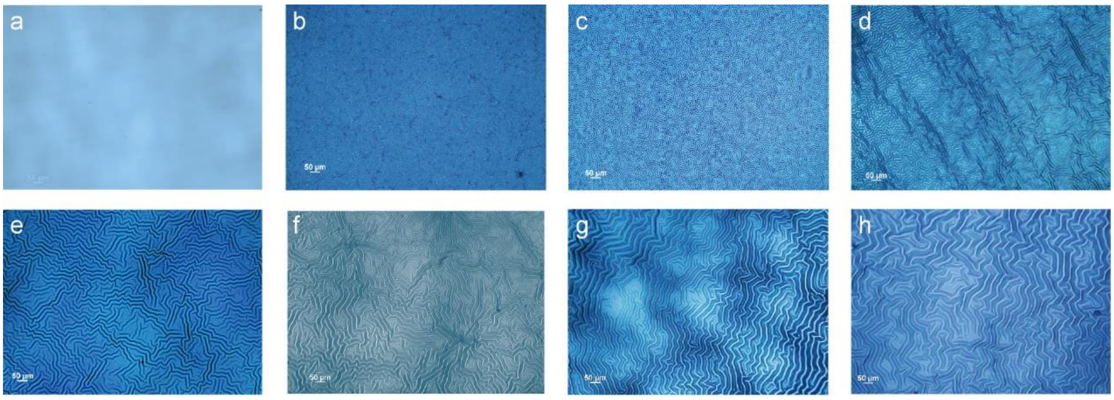

Figure S12. Micro-wrinkles change with substrate modulus (Here, we represent the modulus by the cross-linker ratio). Optical microscope images of parylene micro-wrinkled TSDSs with different characters of micro-wrinkles. These micro-wrinkled TSDSs have the same thickness (3.5 μm) as the top parylene layer. Several sizes of micro-wrinkles can be fabricated by changing the PDSM modulus (here, we use curing ratios to represent their different moduli). **a,** micro-wrinkled parylene-PDMS(10:1); **b,** micro-wrinkled parylene-PDMS(20:1); **c,** micro-wrinkled parylene-PDMS(30:1); **d,** micro-wrinkled parylene-PDMS(40:1); **e,** micro-wrinkled parylene-PDMS(50:1); **f,** micro-wrinkled parylene-PDMS(60:1); **g,** micro-wrinkled parylene-PDMS(70:1); **h,** micro-wrinkled parylene-PDMS(80:1). The scale bar is 50 μm.

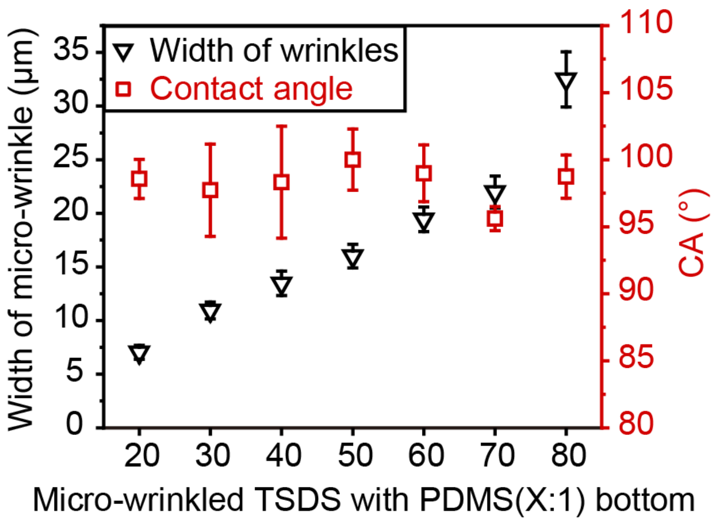

Figure S13. Widths of micro-wrinkles and water contact angle of a series of micro-wrinkled TSDSs. “X:1” means the cross-linker ratio of the PDMS bottom.

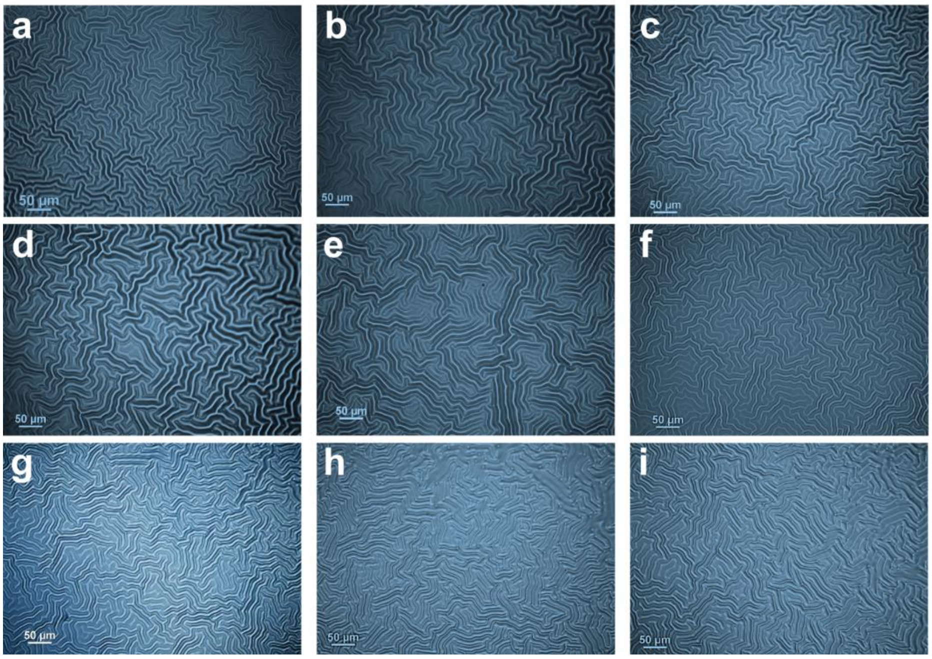

Figure S14. Micro-wrinkles change with parylene thickness. The optical microscope images of parylene micro-wrinkled TSDSs with different top layer thicknesses. PDMS bottom stays in a 50:1 cross-linker ratio. The varied layer thickness is achieved by different masses of input (Figure S4). **a,** 0.1g; **b,** 0.5 g; **c,** 0.7 g; **d,** 1.0 g; **e,** 1.5 g; **f,** 3.0 g; **g,** 5.0 g; **h,** 10.0 g; **i,** 15.0 g. The scale bar is 50 μm. It should be noted that the size of the micro-wrinkles remains largely unchanged across the range of film thicknesses used, which contrasts with traditional wrinkling theory. The wrinkles are probably generated during the early-stage of film deposition, similar to the wrinkle generation by sputtering of metal atoms [1, 2]. In the parylene deposition process, the powdered precursor (dimer) is vaporized under vacuum and heated to form a dimeric gas. This gas is then pyrolyzed to cleave the dimer into its monomeric form, which is subsequently deposited as a transparent polymer film on the PDMS. At the early stages of CVD deposition, the porous nature of PDMS allows the parylene monomer to diffuse into the PDMS polymer during deposition [3]. The modulus and thermal expansion mismatch between parylene and PDMS induce wrinkle formation. Once the wrinkles are generated, their size tends to remain stable during subsequent deposition stages. While an increase in parylene film thickness may slightly reduce the wrinkle width (as parylene fills the wrinkle structure), the wrinkle size is primarily determined by the degree of modulus mismatch between parylene and PDMS, rather than by the film thickness.

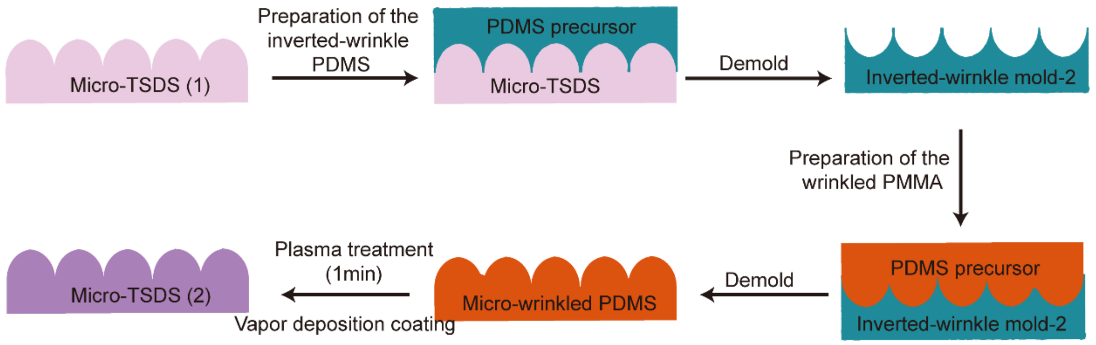

Figure S15. Schematic diagram of the replication process. The process to fabricate the low-modulus micro-wrinkled samples. To eliminate the effects of modulus and film thickness variations. Different numbers of micro-wrinkled samples are fabricated via a molding process (Figure S15). The size of the micro-wrinkles is primarily determined by adjusting the modulus of the PDMS during the molding process. To ensure a constant modulus for the final samples, we used PDMS with a 10:1 curing ratio, which minimizes modulus variability. After molding, these micro-PDMS samples were coated with Parylene C (using the same feed to maintain consistent thickness) following a 1-minute plasma treatment. This process results in micro-TSDS with different sizes of micro-wrinkles

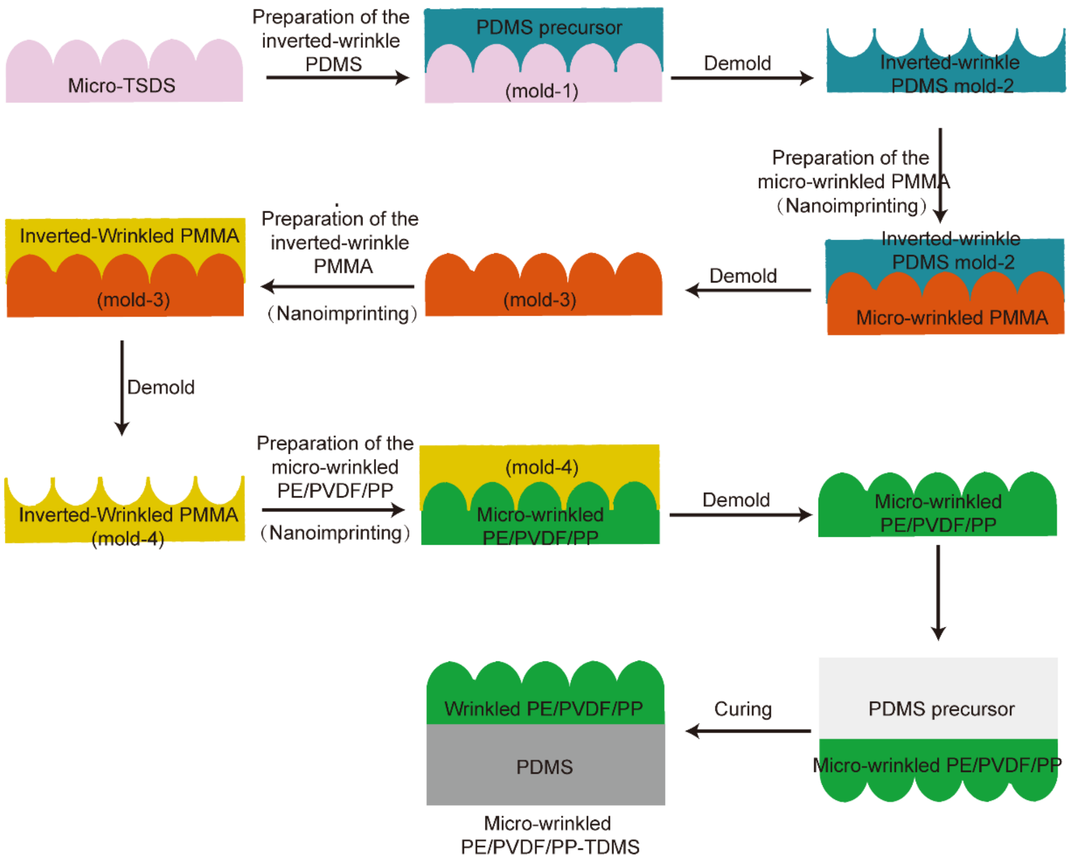

Figure S16. Schematic diagram of the replication process for micro-wrinkled high modulus surface and other types of micro-wrinkled TSDSs. The process of fabricating the high-modulus surface with micro-wrinkled structures and other micro-wrinkled TSDS in various systems.

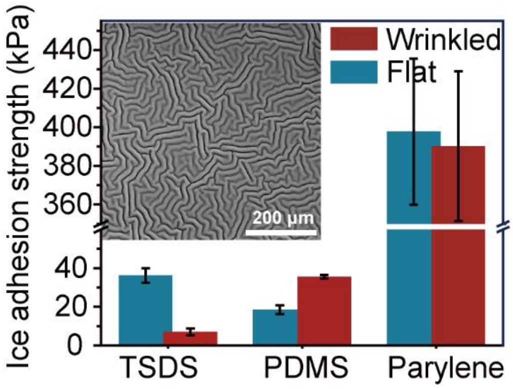

Figure S17. The synergy between microstructure and modulus mismatch. When there is no modulus mismatch (the pure low/high modulus surfaces), the microstructure cannot reduce the adhesion strength and may even increase the adhesion strength. When both exist at the same time, the adhesion strength can be effectively reduced.

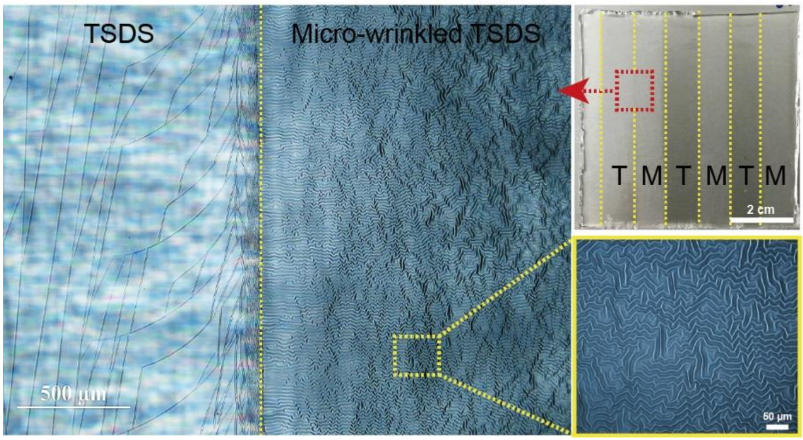

Figure S18. Optical microscope image of TSDS with striped pattern. A TSDS composite with a striped pattern which is composed of the TSDS regions (T) and micro-wrinkled TSDS regions (M).

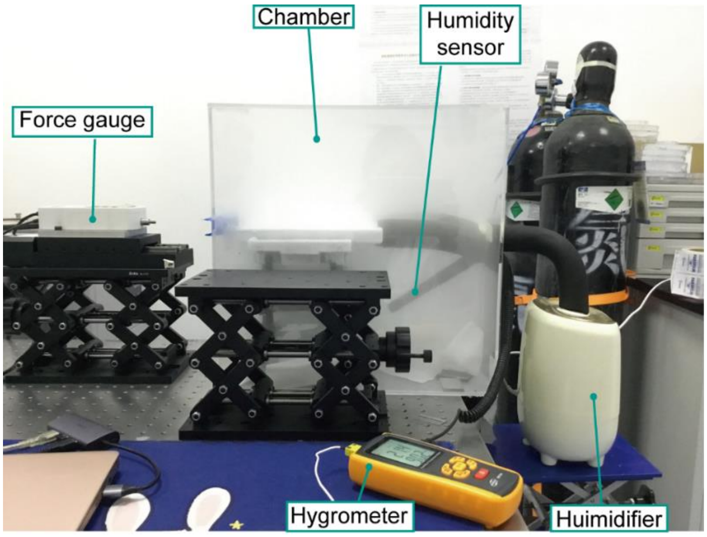

Figure S19. Home-made setup for simulating high humidity freezing environments.

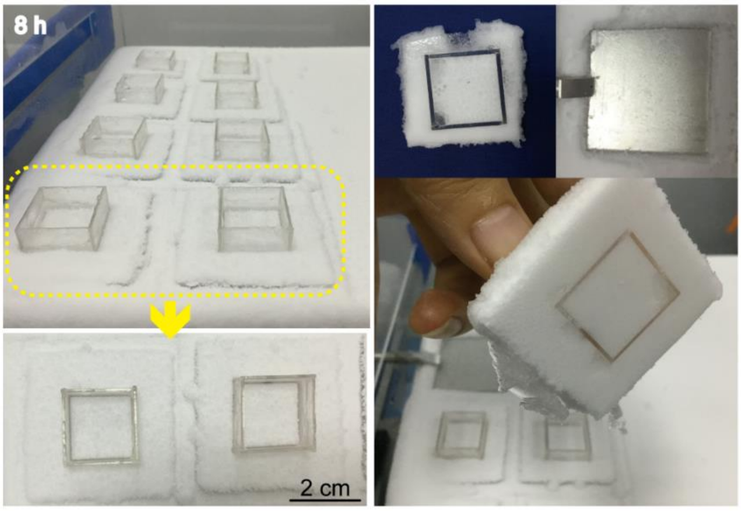

Figure S20. Simulating high humidity freezing environments. Micro-wrinkled TSDSs were covered completely by ice formed from high humidity for 8 hours. The ice is detached easily after applying a small shear force (τ = 6.3±1.2 kPa). The humidity inside the chamber is 93 %.

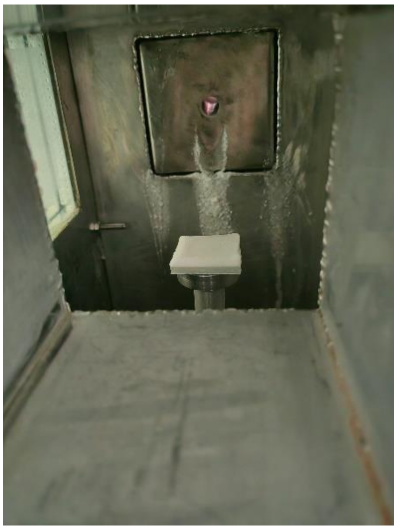

Figure S21. The icing chamber of wind tunnel.

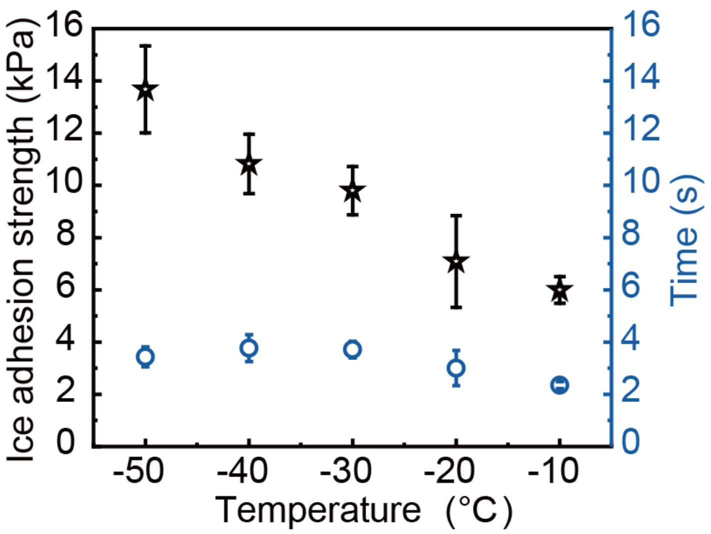

Figure S22. The ice adhesion strength of parylene micro-wrinkled TSDS from -10 ℃ to -50 ℃.

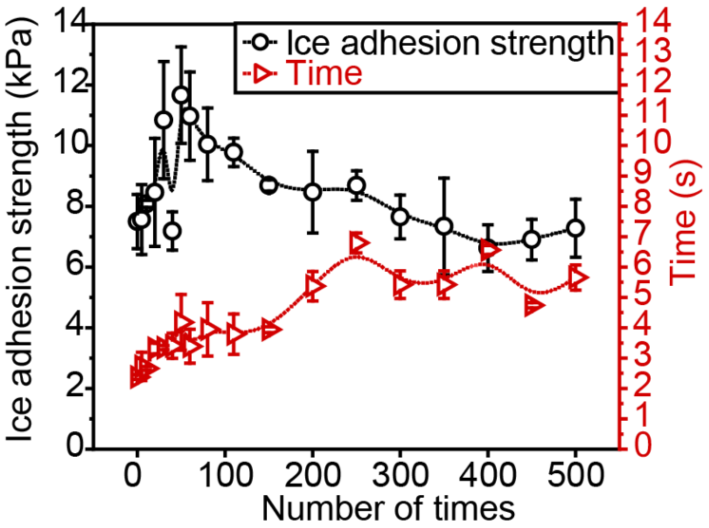

Figure S23. Deicing performance after 500 times tape-peeling tests on the parylene micro-wrinkled TSDS.

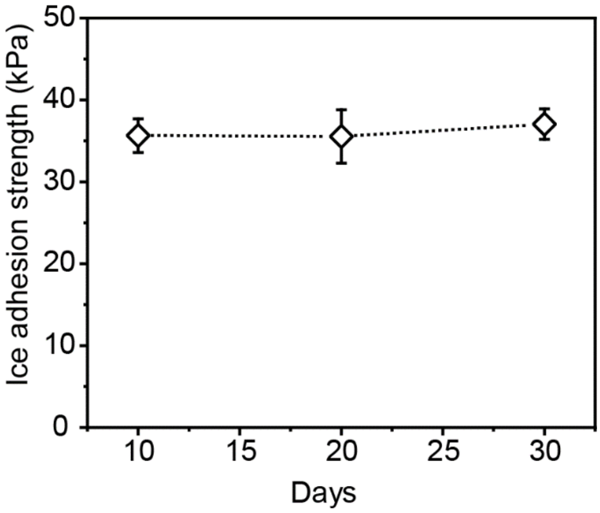

Figure S24. Ultraviolet aging test for 30 days. Put the PVDF micro-wrinkled TSDSs into the UV aging test chamber and perform ice adhesion testing on it after a 10-day cycle.

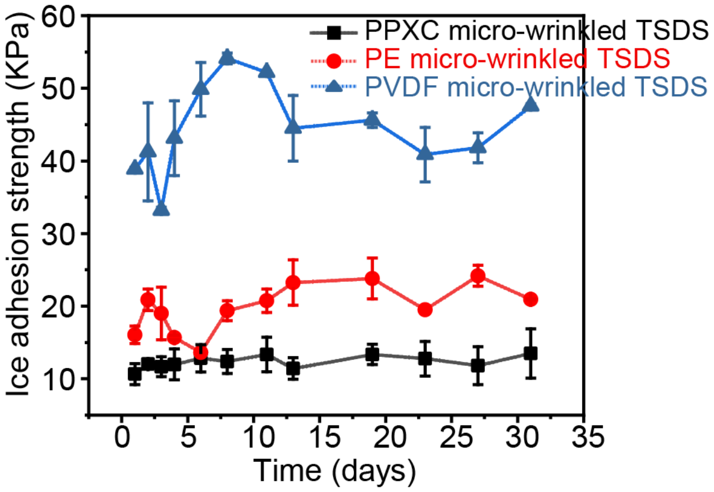

Figure S25. Salt spray test for 31 days. Three types of micro-wrinkled TSDSs were placed in a salt spray testing machine, and after a period of salt spray treatment, the samples were taken out and the surfaces of the samples were cleared before conducting an ice adhesion test. Afterward, put the sample back into the salt spray testing machine again. The salty water concentration is 5 w%.

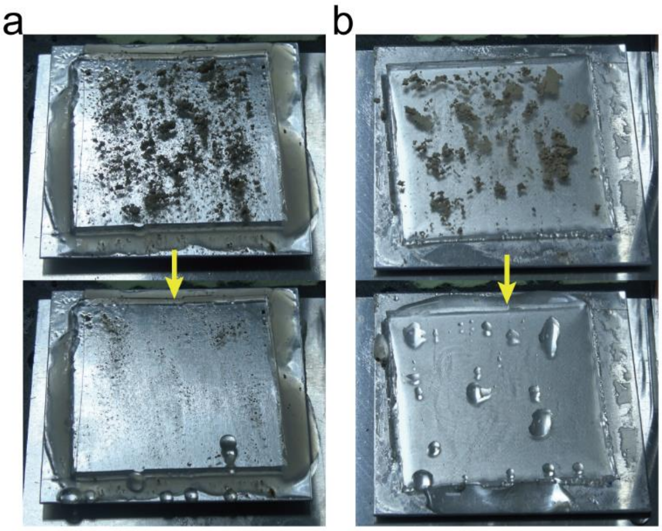

Figure S26. Antifouling test of SPLIS (a) and micro-wrinkled TSDS (b). Dust was scattered on the surfaces of the samples and then rinsed with water. There is still dust residue on the surface of SLIPS, while the dust on the surface of micro-wrinkled TSDS has been completely removed by water.

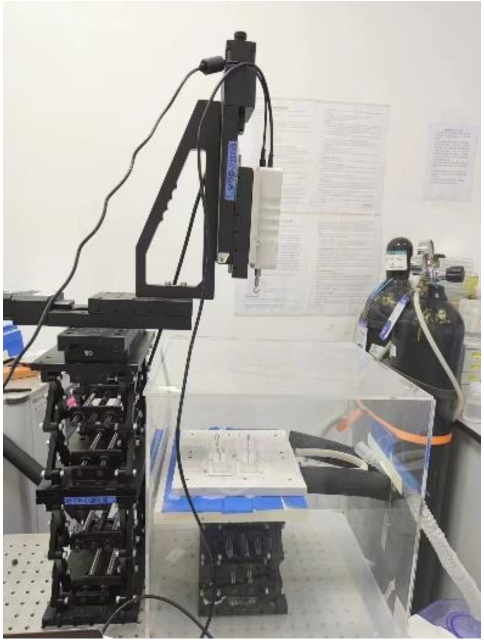

Figure S27. The tensile adhesion test platform and the ice mold.

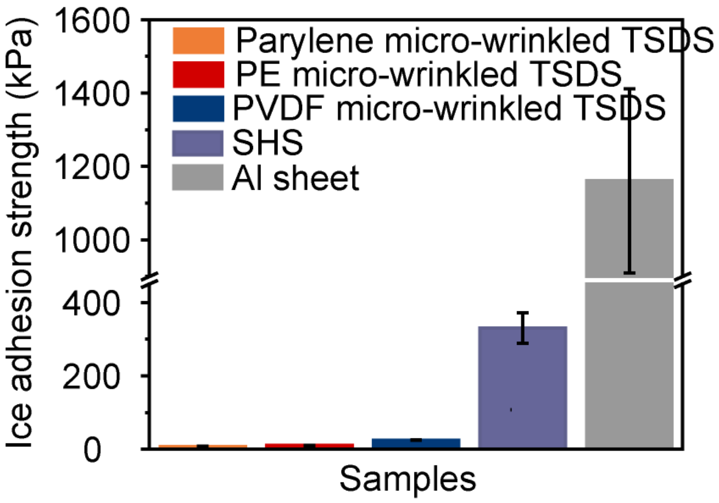

Figure S28. Ice shear adhesion strength for 3 types of surfaces. (micro-wrinkled TSDSs, SHS surface and metal surface). These surfaces are prepared for the simulation test of wind turbine blades.

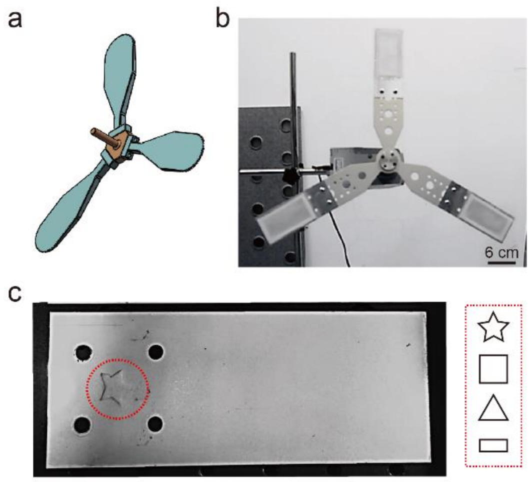

Figure S29. a, Diagram of a simulated wind turbine (a custom rotor). b, The photo of the homemade simulated wind turbine device. The high-speed mixer acts as a speed-adjustable engine. Removable aluminum sheets are mounted on the custom rotor and play the bottom so that the micro-wrinkled TSDSs can be fabricated. c, Different symbols represent different types of micro-wrinkled TSDSs. The pentagram represents a superhydrophobic surface, the rectangle is a bare aluminum sheet. The square represents the PE (10 μm) micro-wrinkled TSDS. The triangle means the PVDF (30 μm) micro-wrinkled TSDS. Nothing is the parylene (3.5 μm) micro-wrinkled TSDS (Movie 6).

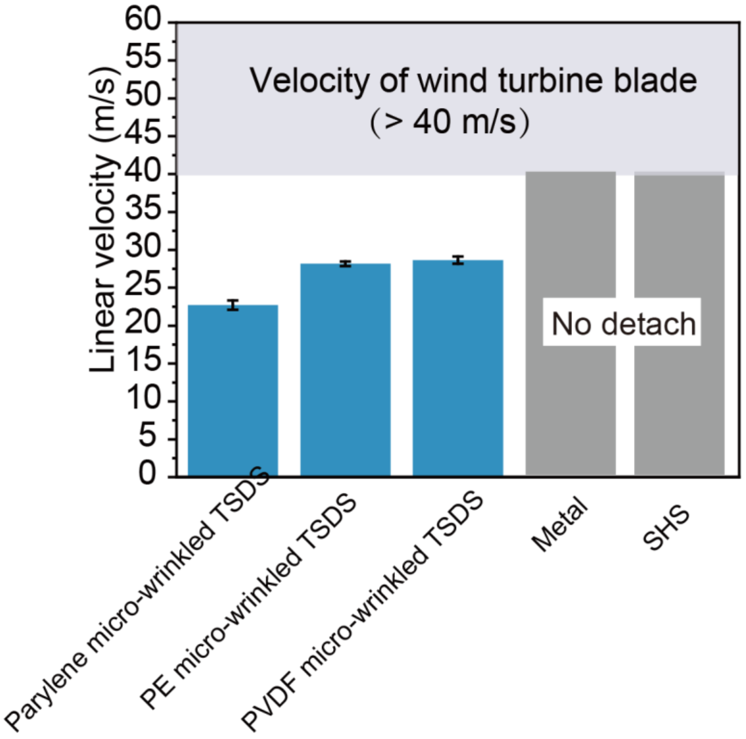

Figure S30. The ice falls off velocity on different surfaces. Modern 600 KW wind turbines (rotor blades about 20m) have blade tip linear speeds ranging from 39.8 to 62.8 m/s. Here, the ice covering the surface of 3 kinds of micro-wrinkled TSDSs can automatically be removed at a small linear speed. When the speed reaches 40 m/s, the SHS and the ice on the metal surface still cannot be separated.

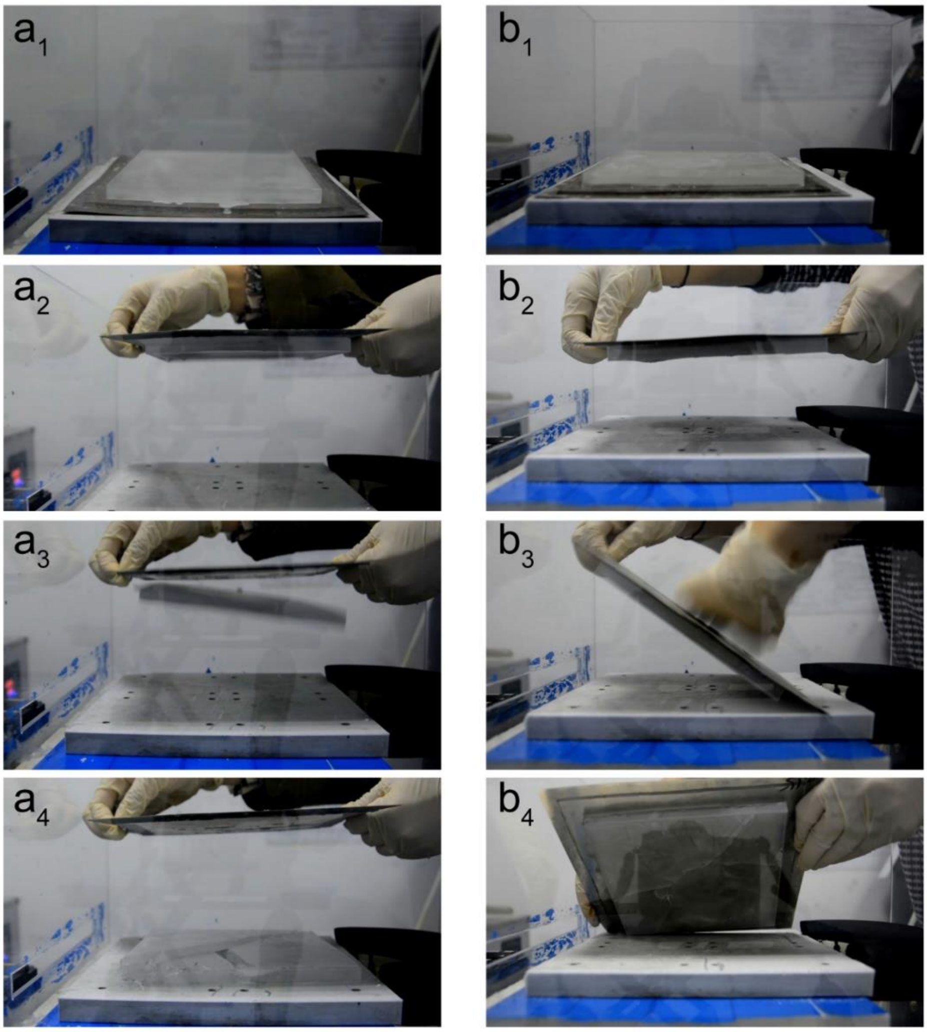

Figure S31. a, The micro-wrinkled TSDS covered a 1cm-thick piece of ice (L =W = 190 mm). Flipping the sheets, the ice falls off due to gravity. b, The ice adhered to PDMS (cross-linker ratio = 50:1) surface cannot remove even turning or patting it.

Supplemental table

Table S1 Peel force and shear force

Shear force (N)	Peel force (N)
142.5 ± 37.85	0.44 ± 0.06

An ice tube (2 × 2 cm^2) was peeled/sheared from a PVDF film with 30 μm thickness.

Table S2 Test conditions for measuring ice adhesion strength

Number	Ice	Temperature (℃)	Wind velocity (m/s)	Water pressure (MPa)	Air pressure (MPa)	Spray time (s)
1	Clear ice	-10	25	0.21	0.33	300
2	Rime ice	-20	25	0.40	0.31	180

Reference

1. Yu S, Sun Y, Ni Y et al. Controlled formation of surface patterns in metal films deposited on elasticity-gradient PDMS substrates. ACS applied materials & interfaces. 2016; 8(8): 5706-5714.
2. Wu K, Yuan H, Li S et al. Two-stage wrinkling of Al films deposited on polymer substrates. Scripta Materialia. 2019; 162: 456-459.
3. Yan S, Zhang J, Chen H et al. Development of a novel magnetophoresis-assisted hydrophoresis microdevice for rapid particle ordering. Biomedical microdevices. 2016; 18: 1-9.
4. SCS Parylene Properties. https://scscoatings.com/parylene-coatings/parylene-properties/
